# Audit: Review of 72-Hour Formulation Meetings on Adult Mental Health Ward

**DOI:** 10.1192/bjo.2026.11808

**Published:** 2026-06-30

**Authors:** Omeno Ohene, Mubeena Sirajudeen, Ijeoma Akuma-Akpu

**Affiliations:** Tees, Esk and Wear Valleys NHS Trust, Darlington, United Kingdom

## Abstract

**Aims:**

**Background:**

Timely multidisciplinary formulation meetings are a cornerstone of patient-centred care in mental health inpatient settings. TEWV NHS Foundation Trust standards recommend completion within 72 hours of admission, with full MDT participation. This audit aimed to evaluate compliance with these standards on Maple Ward, identify delays, and assess attendance patterns to inform quality improvement.

**Methods:**

A retrospective audit was conducted on **67 cases** admitted to Maple Ward between **January and August 2025**. Data were extracted from audit registries and case notes. Variables included admission and formulation dates, attendance by role, and documentation completeness. Descriptive statistics summarized timeliness and attendance. Correlation and linear regression analyses explored associations between delay and team composition.

**Results:**

**Timeliness:** Mean interval from admission to formulation was **4.9 days** (median: 4; range: 1–10 days). Only **27% of cases met the 72-hour standard**, indicating low compliance.

**Attendance:** Consultants (100%), nurses (97%), patients (93%), and resident doctors (91%) were most frequently present. Psychologists (13%) and occupational therapists (25%) attended infrequently; carers were present in 46% of sessions.

**Associations:** Team size showed negligible correlation with delay (r ≈ 0.03). Regression analysis indicated attendance variables did not significantly predict timeliness (Adjusted R² ≈ –0.038; p ≈ 0.71).

**Documentation:** Major gaps included missing documentation of assessment of capacity (91%), legal status (55%), and clinical impression (49%).

**Compliance Level:** 27%.

**Conclusion:**

The audit reveals systemic delays and limited psychosocial input in formulation meetings. Attendance patterns suggest strong core clinical presence but weak representation from psychology and Occupational therapy. Documentation gaps further compromise quality.

**Recommendations**

Improvement strategies include:

- Early scheduling triggers and escalation for delays beyond 72 hours; daily updating of patient board and discussion at report-outs.
- Protected daily formulation slots (Actioned by Ward Manager and Administrative staff)
- Ensure Full involvement and participation of all multidisciplinary team members especially for complex cases.
- Develop a standardized documentation template and induction for resident doctors.

