# Audit: Review of 72-Hour Formulation Meetings on Adult Mental Health Ward – CORRIGENDUM

**DOI:** 10.1192/bjo.2026.12102

**Published:** 2026-09-01

**Authors:** Omeno Ohene, Mubeena Sirajudeen, Ijeoma Akuma-Akpu, Onuwabhagbe Daniel Omomhenle

This abstract was published with an author’s name ommitted. Dr. Onuwabhagbe Daniel Omomhenle has been added as an author and all versions of the article have been updated.

The authors apologise for the error.
